# Eco-friendly synthesis of ionic helical polymers and their chemical properties and reactivity†

**DOI:** 10.1039/c8ra05686b

**Published:** 2018-08-24

**Authors:** Isao Yamaguchi, Yuki Tanaka, Aohan Wang

**Affiliations:** Department of Chemistry, Shimane University 1060 Nishikawatsu Matsue 690-8504 Japan iyamaguchi@riko shimane-u.ac.jp

## Abstract

Reaction of *N*-(2,4-dinitrophenyl)pyridinium chloride (salt(Cl^−^)) with sodium dicyanamide (Na(CN)_2_N) resulted in anion exchange between Cl^−^ and (CN)_2_N^−^ to yield a new Zincke salt, salt((CN)_2_N^−^). Reactions of salt((CN)_2_N^−^) with piperazine, specifically (*R*)-(−)- or (*S*)-(+)-2-methylpiperazine under eco-friendly conditions, such as in aqueous solution, in the absence of a catalyst, and at room temperature, resulted in pyridinium ring opening to yield ionic high-molecular-weight polymers with 5-2,4-dienylideneammonium dicyanamide units or chiral 5-(2-methylpiperazinium)-2,4-dienylideneammonium dicyanamide units, namely, polymer(H;(CN)_2_N^−^), polymer(*R*-Me;(CN)_2_N^−^), and polymer(*S*-Me;(CN)_2_N^−^). UV-Vis measurements revealed that the π-conjugation system expanded along the polymer chain due to the orbital interaction between the electrons on the two nitrogen atoms of the piperazinium ring. Circular dichroism (CD) measurements revealed a helical conformation of the main chain in polymer(*R*-Me;(CN)_2_N^−^) and polymer(*S*-Me;(CN)_2_N^−^). The reaction of polymer(H;(CN)_2_N^−^) with *p*-phenylenediamine (PDA) caused recyclization of the 2,4-dienylideneammonium unit and resulted in depolymerization to yield *N*-(4-aminophenyl)pyridinium dicyanamide. Cyclic voltammetry analysis suggested that the polymers obtained in this study undergo electrochemical oxidation and reduction.

## Abstract 2

Reactions of salt((CN)_2_N^−^) with (*R*)-(−)- or (*S*)-(+)-2-methylpiperazine under eco-friendly conditions resulted in a yield of ionic helical polymers.

## Graphical abstract

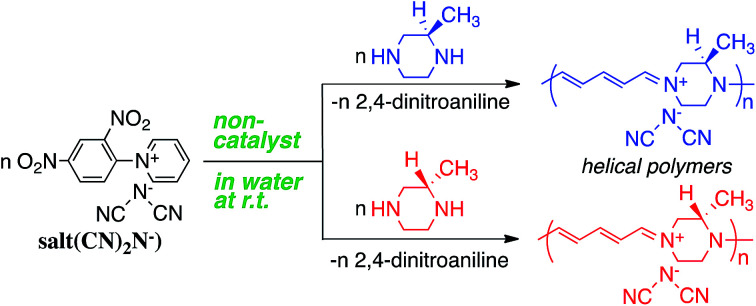

## Introduction

π-Conjugated polymer main chains generally consist of aromatic rings and unsaturated bonds that build an expanded π-conjugation system. The existence of aliphatic cycles in the polymer main chain usually prevents expansion of the π-conjugation system along the polymer chain. We reported the synthesis of ionic polymers—polymer(H;Cl^−^), polymer(*R*-Me;Cl^−^), and polymer(*S*-Me;Cl^−^)—using ring-opening reactions on the pyridinium unit of salt(Cl^−^) with piperazine or chiral 2-methylpiperazines in refluxing ethanol.^1,2^
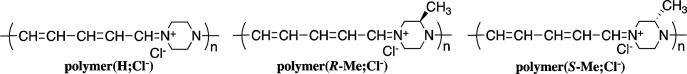


These polymers comprise aliphatic piperazinium rings linked by π-conjugated penta-2,4-dienylideneammonium chloride units. The expanded π-conjugated system along the polymer chain is derived from through-space interactions between the orbitals of nitrogen electrons of the piperazinium ring with the boat form. However, the UV-Vis absorption of the polymers shifted to a shorter wavelength when the solutions were allowed to stand in air. We assumed that the spectral changes were attributed to the decrease in π-conjugation lengths with the spontaneous conversion of piperazinium rings from the boat to the chair form.

The development of non-catalytic syntheses of polymers with expanded π-conjugated system along the polymer chain in water at room temperature is attractive because it offers many advantages with regard to cost, safety, and environmental concerns. The reaction of salt(Cl^−^) with piperazines in water yielded a mixture of unidentified products. We expect that the introduction of a hydrophilic anion into a Zincke salt may enable reaction with an amine in water, and incorporation of electron-accepting groups may contribute to the formation of an intramolecular hydrogen bond with the amine group in the intermediate adduct, which maintains the boat form of the piperazinium ring. Based on this hypothesis, we employed a Zincke salt with a dicyanamide anion, salt((CN)_2_N^−^), in this study. The reaction of salt((CN)_2_N^−^) with piperazines in water without a catalyst at room temperature yielded ionic polymers with high molecular weights. To the best of our knowledge, this is the first report of a non-catalytic synthesis of ionic polymers with expanded π-conjugated system along the polymer chain *via* the reactions under environmentally-friendly conditions. The eco-friendly synthesis of such polymers will lead to new methods for the development of functional materials.

Herein, we report the synthesis of ionic polymers with expanded π-conjugated system along the polymer chain from a monomer with a dicyanamide anion and the chemical structures, reactivity, viscosities, optical properties, and electrochemical properties of the resulting polymers.

## Experimental

### General

Salt(Cl^−^) was prepared according to the literature.^3^ Other reagents were purchased and used without further purification. Solvents were dried, distilled, and stored under N_2_. Reactions were carried out using standard Schlenk techniques under nitrogen.

IR and NMR spectra were recorded on a JASCO FT/IR-660 PLUS spectrophotometer and JEOL AL-400 and ECX-500 spectrometers, respectively. The IR measurement was conducted using a KBr method. UV-Vis and CD spectra were obtained by a JASCO V-560 spectrometer and a JASCO J-720WS, respectively. Multi angle light scattering (MALS) and GPC measurements were conducted with a SHOKO science DAWN HELEOS II and a TOSO HLC-8220 with polystyrene gel columns (Shodex LF-804) using DMF containing 0.06 M LiBr as an eluent, respectively. Cyclic voltammetry was performed on a DMSO solution containing 0.10 M [Et_4_N]BF_4_ with a Hokuto Denko HSV-110. 1 cm × 1 cm and 1 cm × 2 cm Pt plates and Pt wire were used as working, counter, and reference electrodes, respectively.

#### Synthesis of salt((CN)_2_N^−^)

Salt(Cl^−^) (2.82 g, 10.0 mmol) was dissolved in 10 mL of MeOH and sodium dicyanamide (1.07 g, 12.0 mmol) was added to the solution under N_2_. After the solution was stirred at room temperature for 18 h, the precipitate was filtered out, and the filtrate was concentrated under vacuum. The resulting orange solid was extracted with acetone (200 mL). The solvent was removed by evaporation and dried under vacuum to afford salt((CN)_2_N^−^) (3.09 g, 99%). ^1^H NMR (400 MHz, DMSO-*d*_6_): *δ* 9.38 (d, *J* = 6.8 Hz, 2H), 9.13 (t, *J* = 1.6 Hz, 1H), 8.93–9.00 (2H), 8.41–8.46 (3H). ^13^C NMR (125 MHz, DMSO-*d*_6_): *δ* 149.2, 148.9, 146.1, 143.1, 138.8, 131.9, 130.3, 128.1, 121.5, 119.1.

#### Synthesis of polymer(H;(CN)_2_N^−^)

Salt((CN)_2_N^−^) (0.31 g, 1.0 mmol) and piperazine (0.086 g, 1.0 mmol) were dissolved in 3 mL H_2_O under N_2_. After the solution was stirred at room temperature for 18 h, the precipitate was collected by filtration, washed with acetone (200 mL) and dried *in vacuo* to afford polymer(H;(CN)_2_N^−^) (0.35 g, 71%). ^1^H NMR (400 MHz, DMSO-*d*_6_): *δ* 7.92 (2H), 7.56 (1H), 6.03 (2H), 3.55, 3.81 (8H).

Other polymers were synthesized in a similar manner.

#### Polymer(*rac*-Me;(CN)_2_N^−^)

Yield = 21%. ^1^H NMR (400 MHz, DMSO-*d*_6_): *δ* 7.92 (2H), 7.58 (1H), 6.07 (2H), 3.57–4.17 (7H), 1.31 and 1.22 (3H).

#### Polymer(*R*-Me;(CN)_2_N^−^)

Yield = 34%. ^1^H NMR (400 MHz, DMSO-*d*_6_): *δ* 7.92 (2H), 7.57 (1H), 6.07 (1H), 3.57–4.16 (7H), 1.31 and 1.22 (3H).

#### Polymer(*S*-Me;(CN)_2_N^−^)

Yield = 36%. ^1^H NMR (400 MHz, DMSO-*d*_6_): *δ* 7.93 (2H), 7.58 (1H), 6.10 (2H), 3.57–4.15 (7H), 1.31 and 1.22 (3H).

#### Synthesis of model compound

Salt((CN)_2_N^−^) (0.31 g, 1.00 mmol) was dissolved in 4 mL water and 1-phenylpiperazine (0.32 g, 2.0 mmol) was added to the solution under N_2_. The solution was stirred for 18 h at room temperature. The resulting precipitate was collected by filtration, washed with ether twice and dried *in vacuo* to afford model((CN)_2_N^−^) (0.28 g, 74%). ^1^H NMR (400 MHz, DMSO-*d*_6_): *δ* 7.90 (d, *J* = 12.0 Hz, 2H), 7.51 (t, *J* = 12.4 Hz, 1H), 7.24–7.28 (m, 4H), 7.00 (d, *J* = 8.0 Hz, 4H), 6.84 (t, *J* = 7.2 Hz, 2H), 6.05 (t, *J* = 12.4 Hz, 2H), 3.73 and 3.76 (8H), 3.32 (8H). ^13^C NMR (125 MHz, DMSO-*d*_6_): *δ* 163.0, 160.0, 150.0, 129.0, 119.6, 116.0, 102.8, 53.3, 48.7, 47.6, 45.8. ESI TOF-MS: calcd for C_25_H_31_N_4_ (cation), 387.2543. Found, 387.2560.

#### Reaction of polymer(H;(CN)_2_N^−^) with PDA

After the DMSO solution (3 mL) of polymer(H;(CN)_2_N^−^) (0.43 g, 2.0 mmol) and PDA (0.22 g, 2.0 mmol) was stirred at room temperature for 72 h, the precipitate from the solution was removed by filtration, and the filtrate was concentrated under vacuum. The resulting paste was extracted with methanol (200 mL), the solvent was removed by evaporation and dried *in vacuo* to yield *N*-(4-aminophenyl)pyridinium dicyanamide (APD) (0.34 g, 71%). ^1^H NMR (400 MHz, DMSO-*d*_6_): *δ* 9.17 (d, *J* = 6.0 Hz, 2H), 8.62 (t, *J* = 8.0 Hz, 1H), 8.20 (d, *J* = 6.8 Hz, 2H), 7.49 (d, *J* = 7.2 Hz, 2H), 6.75 (d, *J* = 8.8 Hz, 2H), 5.90 (s, 2H). ESI TOF-MS calcd for C_13_H_11_N_4_ (cation): 171.0917. Found: 171.0924.

#### Reaction of model(H;(CN)_2_N^−^) with PDA

After the acetone solution (3 mL) of model(H;(CN)_2_N^−^) (0.045 g, 0.10 mmol) and PDA (0.011 g, 0.10 mmol) was stirred at room temperature for 48 h, the solvent was removed under vacuum. The ^1^H NMR and ESI TOF-MS measurements suggested that the resulting solid contains APD and 1-(4-aminophenyl)piperazine (APP). ^1^H NMR data of APP (400 MHz, DMSO-*d*_6_): *δ* 6.89–6.96 (m, 5H), 3.12 (t, *J* = 4.8 Hz, 1H), 3.02 (t, *J* = 4.8 Hz, 4H), 2.83 (t, *J* = 5.2 Hz, 4H). ESI TOF-MS: calcd for C_13_H_11_N_4_ (cation of APD), 171.0917; [APP + H^+^], 163.1235. Found, 171.0923, 163.1234.

## Results and discussion

### Synthesis

**Scheme 1:**
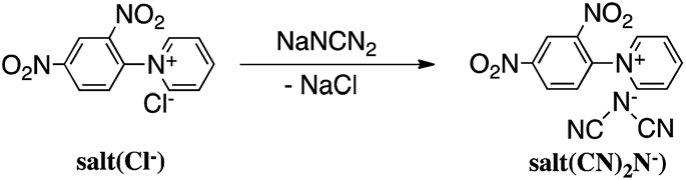
Synthesis of monomer.

**Scheme 2:**
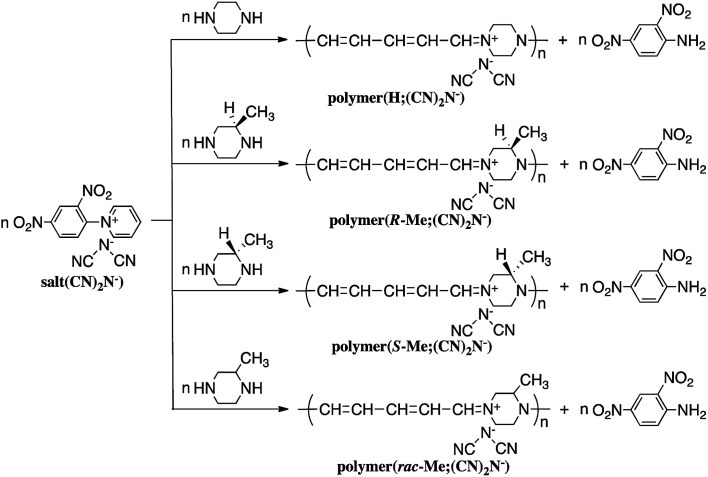
Synthesis of polymers.

Reaction of *N*-(2,4-dinitrophenyl)pyridinium chloride (salt(Cl^−^)) with sodium dicyanamide resulted in anion exchange between Cl^−^ and (CN)_2_N^−^ to yield *N*-(2,4-dinitrophenyl)pyridinium dicyanamide (salt((CN)_2_N^−^)) (Scheme 1). The reactions of salt((CN)_2_N^−^) with piperazine, *R*-(−)-2-methylpiperazine, *S*-(+)-2-methylpiperazine and 2-methylpiperazine in water at room temperature led to ring-opening of the pyridinium ring to afford polymer(H;(CN)_2_N^−^), polymer(*R*-Me;(CN)_2_N^−^), polymer(*S*-Me;(CN)_2_N^−^) and polymer(*rac*-Me;(CN)_2_N^−^), in 71%, 34%, 36% and 21% yields, respectively (Scheme 2). However, the reaction of salt(Cl^−^) with piperazine in water at room temperature resulted in unidentified products.

**Scheme 3:**
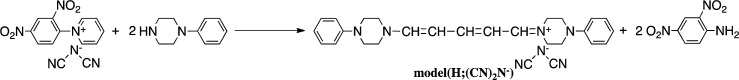
Synthesis of model compound.

The model compound model(H;(CN)_2_N^−^) was synthesized by the reaction of 1-phenylpiperazine with salt((CN)_2_N^−^) in 74% yield (Scheme 3). The results of these reactions are summarized in Table 1.

**Table 1:** Synthesis results and properties of polymers and model compound

Product	Yield, %	*η*_sp_/*c*^a^, g^−1^ dL	*M*_w_ b	Absorption^c^, nm	*E*_a_ d, V	*E*_c_ d, V
Polymer(H;(CN)_2_N^−^)	71	0.78	53.2 × 10^4^	472	0.67	—f
Polymer(*R*-Me;(CN)_2_N^−^)	34	0.62	3.95 × 10^4^	473	0.71	−0.60
						−1.34
Polymer(*S*-Me;(CN)_2_N^−^)	36	0.60	1.16 × 10^4^	472	0.70	−0.61
						−1.35
Polymer(*rac*-Me;(CN)_2_N^−^)	21	0.56	13.7 × 10^4^	480	0.72	−0.61
						−1.35
Model((CN)_2_N^−^)	74			424	1.32^e^	−0.70
						−1.36^e^

a In DMSO at 30 °C.

b Determined by MALS.

c In DMSO.

d Measured by cyclic voltammetry. Cast film of the polymer on a Pt plate in an acetonitrile solution of [Et_4_N]BF_4_ (0.1 M). Sweep rate was 50 mV s^−1^.

e In an acetonitrile solution of [Et_4_N]BF_4_ (0.1 M).

f Not observed.

The polymers obtained in this study were completely soluble in polar organic solvents such as *N*,*N*-dimethylformamide (DMF), dimethyl sulfoxide (DMSO) and *N*-methyl-2-pyrrolidone (NMP). Polymer(*rac*-Me;(CN)_2_N^−^), polymer(*R*-Me;(CN)_2_N^−^) and polymer(*S*-Me;(CN)_2_N^−^) were partially soluble in methanol but polymer(H;(CN)_2_N^−^) was insoluble in the solvent. Model(H;(CN)_2_N^−^) was soluble in acetone, MeOH, DMF and DMSO. The weight-averaged molecular weights (*M*_w_'s) of NMP solutions of polymer(H;(CN)_2_N^−^), polymer(*R*-Me;(CN)_2_N^−^) and polymer(*rac*-Me;(CN)_2_N^−^), as determined by light scattering method, were 53.2 × 10^4^, 3.95 × 10^4^ and 13.7 × 10^4^, respectively. The *M*_w_ value of polymer(*R*-Me;(CN)_2_N^−^), as determined by GPC, was 3.27 × 10^4^ (*M*_w_/*M*_n_ = 1.46).

The reduced viscosities (*η*_sp_/*c*) of the DMSO solutions of the polymers are summarized in Table 1. The *η*_sp_/*c* values of the polymers in DMSO increased as their concentrations, *c*, were reduced (Fig. S1†). The *η*_sp_/*c* values of polymer(H;(CN)_2_N^−^), polymer(*R*-Me;(CN)_2_N^−^) and polymer(*rac*-Me;(CN)_2_N^−^) changed from 0.78, 0.62 and 0.56 g^−1^ dL (*c* = 0.10 g dL^−1^) to 0.95, 0.74 and 0.71 g^−1^ dL (*c* = 0.050 g dL^−1^) through values of 0.85, 0.66 and 0.64 g^−1^ dL (*c* = 0.071 g dL^−1^). These results suggest that the polymers behaved as polymeric electrolytes in dilute solutions.^4^ The *η*_sp_/*c* values of polymer(*R*-Me;(CN)_2_N^−^) and polymer(*rac*-Me;(CN)_2_N^−^) were roughly the same, although the *M*_w_ value of polymer(*rac*-Me;(CN)_2_N^−^) was considerably higher than that of polymer(*R*-Me;(CN)_2_N^−^). The results imply that polymer(*R*-Me;(CN)_2_N^−^) has a stiffer structure than polymer(*rac*-Me;(CN)_2_N^−^) in solution. It has been reported that helical polymers show greater reduced viscosities due to their stiff structures when compared to random coil polymers.^5^

### NMR and IR spectra

**Fig. 1:**
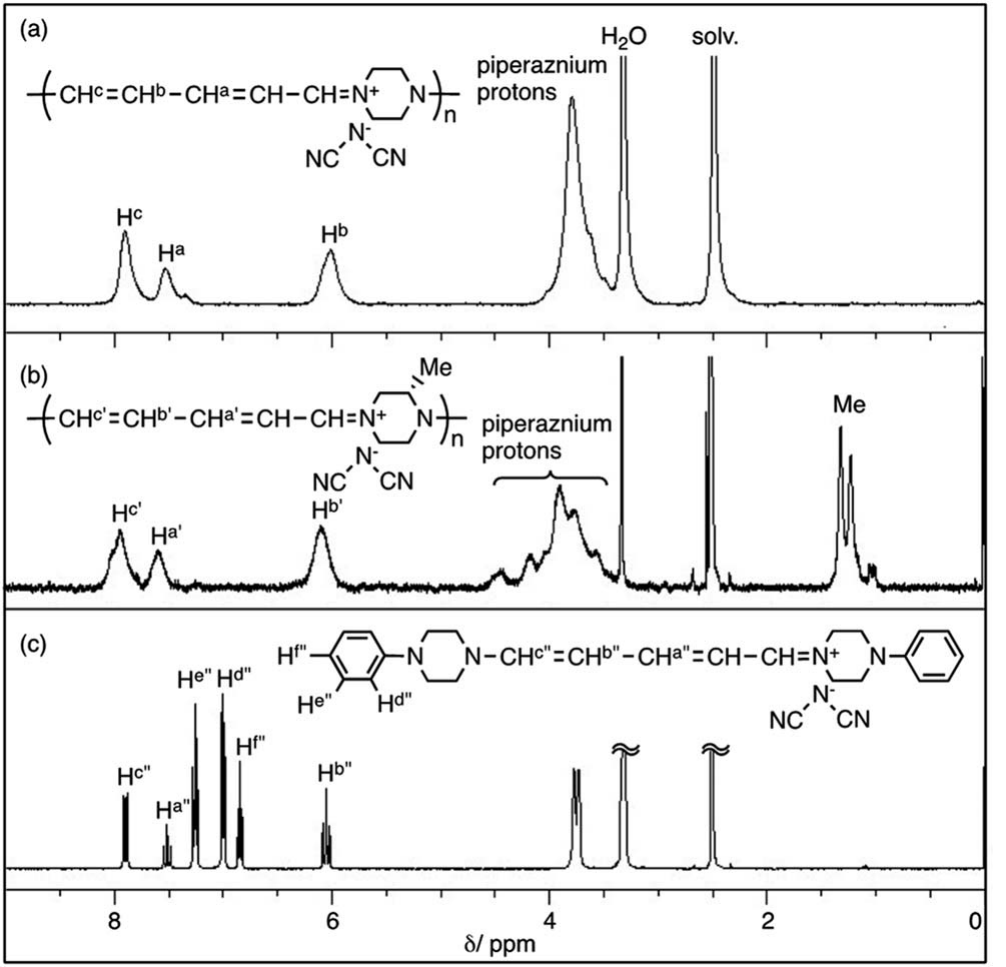
^1^H NMR spectra of polymer(H;(CN)_2_N^−^), polymer(*S*-Me;(CN)_2_N^−^) and model(H;(CN)_2_N^−^) in DMSO-*d*_6_.

Fig. 1 shows the ^1^H NMR spectra of polymer(H;(CN)_2_N^−^), polymer(*S*-Me;(CN)_2_N^−^) and model(H;(CN)_2_N^−^) in DMSO-*d*_6_. The peak assignments are shown in the figure. The ^1^H NMR spectra of the DMSO-*d*_6_ solutions of polymer(*R*-Me;(CN)_2_N^−^) and polymer(*S*-Me;(CN)_2_N^−^) were similar, and each spectrum showed three signals, at approximately *δ* 7.9, 7.6, and 6.1 in a 2 : 1 : 2 integral ratio, arising from the protons in the penta-2,4-dienylideneammonium group. The ^1^H NMR spectra of the DMSO-*d*_6_ solution of polymer(H;(CN)_2_N^−^) also showed three signals corresponding to the penta-2,4-dienylideneammonium group at almost the same positions corresponding those of polymer(*S*-Me;(CN)_2_N^−^) in a 2 : 1 : 2 integral ratio, arising from the protons in the penta-2,4-dienylideneammonium group. These observations suggest that the π-electrons are delocalized along the penta-2,4-dienylideneammonium group. The integral ratio of the peaks corresponding to the methyl group and piperazinium protons of the polymers supports the structures shown in Scheme 1. The presence of three signals corresponding to hydrogen atoms H^c^′′, H^b^′′ and H^a^′′ of model(H;(CN)_2_N^−^) in a 2 : 2 : 1 integral ratio suggests that the π-electrons are delocalized across the aminopenta-2,4-dienylidene group. The ^13^C NMR data also support this view, showing three signals arising from the penta-2,4-dienylideneammonium group (Fig. S2†).

### Reactivity

**Scheme 4:**
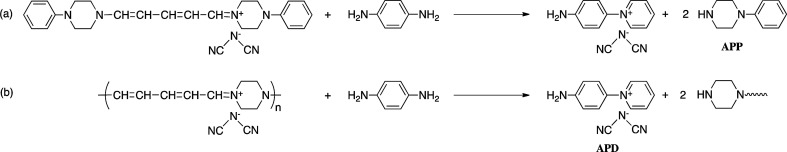
Recylization reactions.

The reaction of model(H;(CN)_2_N^−^) with PDA caused recyclization of the 2,4-dienylideneammonium unit and resulted in the formation of *N*-(4-aminophenyl)pyridinium dicyanamide (APD) and *N*-phenylpiperazine (Scheme 4a). The formation of these compounds was confirmed by their ESI TOF-MS and ^1^H NMR spectra (Fig. S3 and S4†). The reaction of polymer(H;(CN)_2_N^−^) with PDA also caused recyclization of the 2,4-dienylideneammonium unit of the polymer and resulted in depolymerization to yield APD (Scheme 4b).

**Scheme 5:**
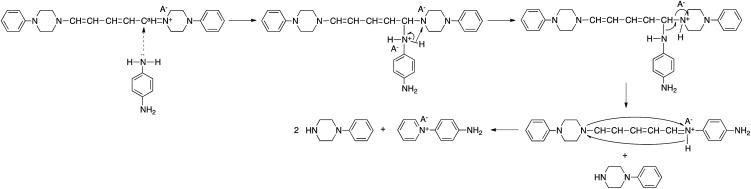
Possible reaction mechanism.

It has been reported that 5-anilino-*N*-phenyl-2,4-pentadienylideniminium chloride is converted spontaneously and quantitatively to *N*-phenylpyridinium chloride and aniline in solution.^3^ However, the ring-closure of the 2,4-pentadienylideniminium group by reaction with an amine has not been previously reported. The assumption that the above reactivity is due to the presence of dicyanamide anions in the polymer and model compound is confirmed by the result that polymer(H;Cl^−^) and model(H;Cl^−^) did not react with PDA. Pyridinium salts are an important class of compounds that are used as initiators of cationic polymerization,^8^ cationic surfactants,^9^ non-linear optical materials,^10^ and phase transfer catalysts.^11^ The reactive pyridinium salts could be used as starting species for functional materials such as dyes, nonlinear optical polymers, and polymer catalysts. However, reports on such reactive pyridinium salts are limited because of difficulty in synthesis of them.^12^ In fact, APD could not be obtained by the reaction of salt(Cl^−^) with PDA. In contrast, the ring-closure reaction yielded APD. Scheme 5 shows a possible reaction mechanism for the recyclization of the 2,4-dienylideneammonium unit of model(H;(CN)_2_N^−^). Nucleophilic addition of one of the amine groups of PDA to the carbon atom (C^a^) bonded to the piperazinium ring occurs first. The fact that the electron density of C^a^ is lowest among the 2,4-dienylideneammonium carbons is confirmed by the appearance of the ^1^H NMR signal at the lowest magnetic field position among the 2,4-dienylideneammonium protons.

The newly formed C–N bond exchanges between the aminophenyl and piperazinium rings, followed by the ring-closure of the 5-piperizine-*N*-(4-aminophenyl)-2,4-pentadienylideniminium unit to yield *N*-(4-aminophenyl)pyridinium dicyanamide and *N*-phenylpiperazine. The reaction of *N*-(2,4-dinitrophenyl)penta-2,4-dienylidene-1-*N*-arylinium chloride with piperizine to provide *N*-(5-piperidino-2,4-pentadienylidene)piperizinium chloride has also been previously reported.^6,7^

**Scheme 6:**
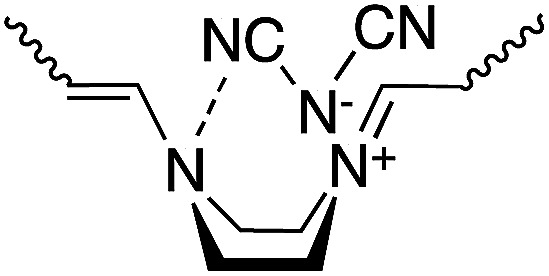
Intramolecular hydrogen bond between the cyano group and the nitrogen atom.

It is known that piperazine derivatives usually assume the energetically stable chair form rather than the boat form.^13^ However, the piperazinium and 2-methylpiperazinium rings of the polymers obtained in this study primarily assume the boat form, which contributes the expansion of π-conjugation system, as revealed by the following UV-Vis measurements. The intramolecular hydrogen bond between the nitro group and the NH group in the intermediate adduct seems to play an important role in the predominant formation of the piperazinium ring with the boat form in the polymers, as illustrated in Scheme S1.† The piperazinium and 2-methylpiperazinium rings of polymer(H;Cl^−^), polymer(*S*-Me;Cl^−^) and polymer(H;Cl^−^) converted from the boat to chair form when solutions of each were allowed to stand in air.^1,2^ The possibility that the UV-Vis spectral changes were attributed to decomposition of the polymers could be ruled out from the result that the viscosities of the polymer solutions did not change with time. Whereas such interconversion of the piperazinium and 2-methylpiperazinium rings of the polymers with dicyanamide anions was not observed. In other words, the polymers can remain their extended π-conjugation system in solution. We speculated that this result may be the intramolecular interaction between the nitrogen atom of the piperazinium ring in the boat form and a CN group of the dicyanamide anion, as illustrated in Scheme 6.

### UV-Vis spectra

**Fig. 2:**
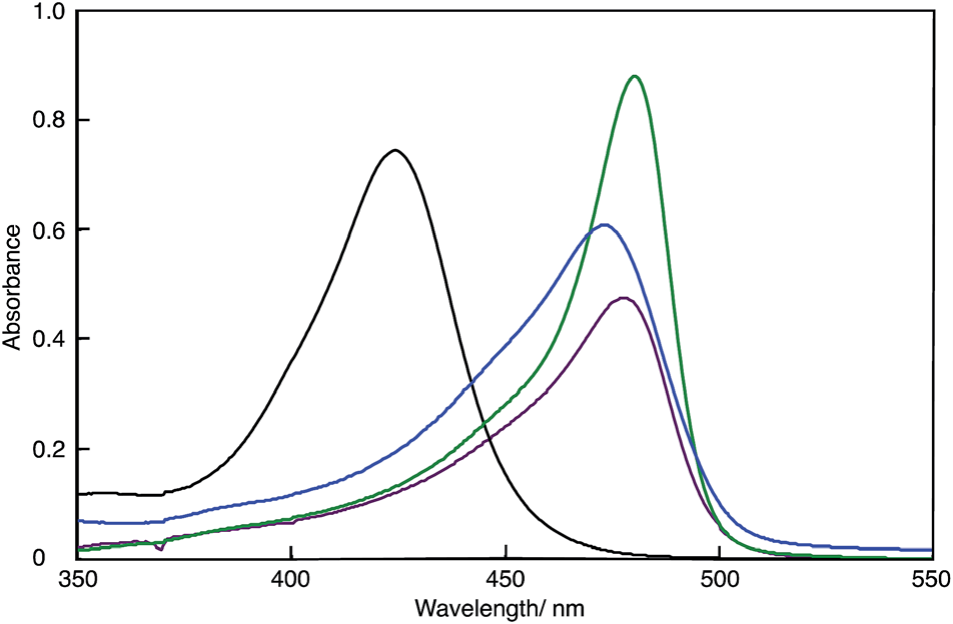
UV-Vis spectra of polymer(*H*-Me;(CN)_2_N^−^) (purple curve), polymer(*S*-Me;(CN)_2_N^−^) (blue curve), polymer(H;(CN)_2_N^−^) (green curve) and model(H;(CN)_2_N^−^) (black curve) in DMSO (*c* = 1 × 10^−5^ M^−1^).

Fig. 2 shows the UV-Vis spectra of the DMSO solutions of polymer(*H*-Me;(CN)_2_N^−^), polymer(*S*-Me;(CN)_2_N^−^), polymer(*rac*-Me;(CN)_2_N^−^) and model(H;(CN)_2_N^−^). The UV-Vis data are summarized in Table 1. The absorption maxima (*λ*_max_) of the DMSO solutions of the polymers appeared at longer wavelengths than did that of model(H;(CN)_2_N^−^), suggesting extension of π-conjugation across the aminopenta-2,4-dienylidene groups and through the 2-methylpiperazinium and piperazinium rings. The *λ*_max_'s of polymer(*H*-Me;(CN)_2_N^−^) in DMF and methanol were 473 and 475 nm, respectively. These wavelengths were essentially the same as that in DMSO. The change in the UV-Vis spectra over time of freshly prepared MeOH solutions of polymer(*R*-Me;Cl^−^), polymer(*S*-Me;Cl^−^) and polymer(H;Cl^−^) at 30 °C in air corresponds to the two-step conversion of 2-methylpiperazinium and piperazinium rings from the boat to the chair form *via* a half-chair intermediate.^1,2^ When the 2-methylpiperazinium rings of the polymers adopt the chair conformation, the distance between its two nitrogen atoms is too large to allow through-space interaction; therefore, there is no expanded π-conjugation in these polymers. We proposed a possible reaction mechanism for the predominant formation of the thermodynamically unstable boat form of piperazinium ring in the polymers at the initial stage.^1^ However, the UV-Vis spectra of solutions of polymer(*R*-Me;(CN)_2_N^−^), polymer(*S*-Me;(CN)_2_N^−^) and polymer(H;(CN)_2_N^−^) in DMSO were almost unchanged when the solutions were allowed to stand in air. The UV-Vis spectra of DMF and methanol solutions of polymer(H;(CN)_2_N^−^) were also unchanged when the solutions were allowed to stand in air. This result suggests that the 2-methylpiperazinium and piperazinium rings did not change their conformations in solutions and maintained the π-conjugation lengths of the polymers.

The absorption of the DMSO solution of polymer(H;(CN)_2_N^−^) at 472 nm in the presence of an equimolar amount of PDA decreased with time when the solution was left open to air (Fig. S6†). The decrease in this absorbance corresponds to the formation of APD, accompanied by depolymerization of the polymer.

### CD spectra

**Fig. 3:**
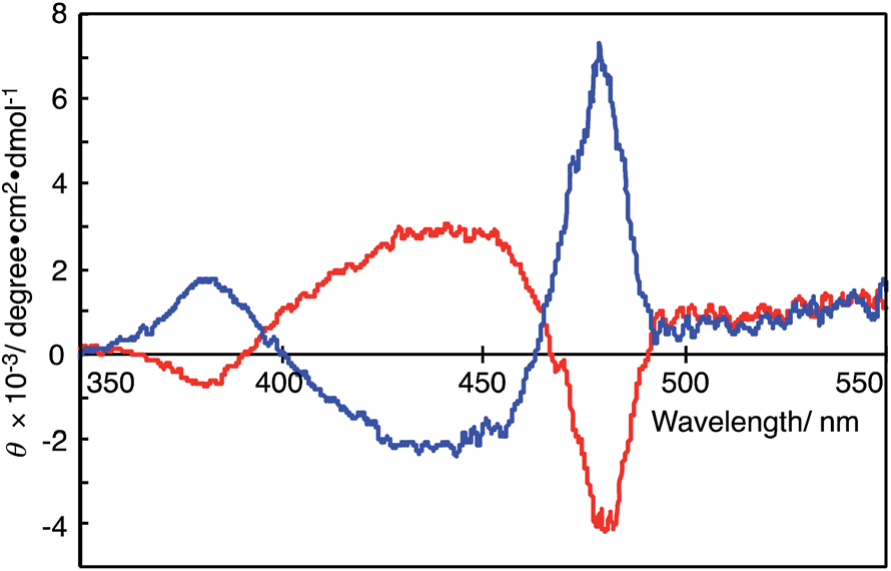
CD spectra of polymer(*R*-Me;(CN)_2_N^−^) (blue curve) and polymer(*S*-Me;(CN)_2_N^−^) (red curve) in DMSO (*c* = 1.0 × 10^−5^ M^−1^).

Fig. 3 shows the CD spectra of the DMSO solutions of polymer(*R*-Me;(CN)_2_N^−^) and polymer(*S*-Me;(CN)_2_N^−^). The CD spectrum of polymer(*R*-Me;(CN)_2_N^−^) displays a relatively strong positive Cotton effect, with zero-crossings centered at the π–π* transition of the polymer chain (at approximately 470 nm in the UV-Vis absorption spectra, see Fig. 2). The positive Cotton effect in the CD spectrum of polymer(*R*-Me;(CN)_2_N^−^) is consistent with a similar trend for the polymer(*R*-Me;Cl^−^).^2^ Similarly, polymer(*S*-Me;(CN)_2_N^−^) showed a relatively strong negative Cotton effect. These Cotton curves suggest that the main chains of polymer(*R*-Me;(CN)_2_N^−^) and polymer(*S*-Me;(CN)_2_N^−^) have oppositely oriented, highly ordered structures, such as right- and left-handed helical conformations. The ellipticities for the Cotton curves of polymer(*R*-Me;Cl^−^) and polymer(*S*-Me;Cl^−^) gradually decreased with time when the methanol solutions were allowed to stand in air. These decreases are presumably due to time-dependent dynamic conformational changes in the polymers; the helical conformation of the polymers disappeared as the 2-methylpiperazinium ring converted from the boat to the chair form *via* a half-chair intermediate form. Whereas such decrease in ellipticities for the Cotton curves was not observed in the CD spectra of polymer(*R*-Me;(CN)_2_N^−^) and polymer(*S*-Me;(CN)_2_N^−^). The results are consistent with the fact that the UV-Vis spectra of the DMSO solutions of the polymers were almost unchanged with time.

### Cyclic voltammograms

**Fig. 4:**
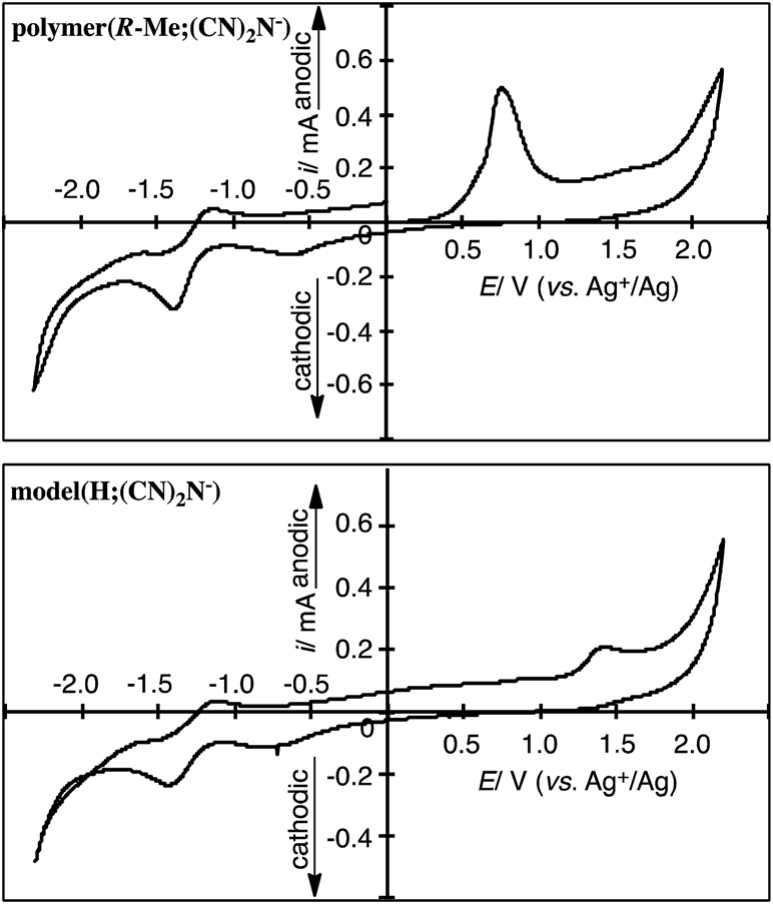
CV curves of the cast film of polymer(*R*-Me;(CN)_2_N^−^) on a Pt plate in the DMSO solution of [Et_4_N]BF_4_ (0.1 M) and model(H;(CN)_2_N^−^) in the DMSO solution (1 mM) of [Et_4_N]BF_4_.

Cyclic voltammetry (CV) measurements suggest that the polymers and model compound obtained in this study underwent electrochemical oxidation of the 2-methylpiperazinium and piperazinium rings and reduction of the dicyanamide anions. The oxidation and reduction potentials of the polymers and model compound in DMSO containing 0.10 M [Et_4_N]BF_4_ are summarized in Table 1. Fig. 4 shows the CV curves of polymer(*R*-Me;(CN)_2_N^−^) and model(H;(CN)_2_N^−^). Polymer(*R*-Me;(CN)_2_N^−^) and model(H;(CN)_2_N^−^) underwent electrochemical oxidation of the piperazinium ring and reduction of the CN groups of the dicyanamide anion. The oxidation peak potential of polymer(*R*-Me;(CN)_2_N^−^) was lower (*E*_pa_ = 0.71 V *vs.* Ag^+^/Ag) than that of model(H;(CN)_2_N^−^) (*E*_pa_ = 1.32 V *vs.* Ag^+^/Ag) because of the longer π-conjugation length of the polymer. The oxidation peak potential of polymer(*S*-Me;(CN)_2_N^−^) was the essentially same as that of polymer(*R*-Me;(CN)_2_N^−^). The dicyanamide anions of the polymers, except for polymer(H;(CN)_2_N^−^), and the model compound participated in two-step electrochemical reductions at approximately −0.6 V and −1.3 V (*vs.* Ag^+^/Ag). The reason for the disappearance of the reduction peaks in polymer(H;(CN)_2_N^−^) is unclear.

## Conclusions

In conclusion, ionic helical polymers were obtained from the reaction of *N*-(2,4-dinitrophenyl)pyridinium dicyanamide with chiral 2-methylpiperazines in water at room temperature. UV-Vis measurements revealed that the 2-methylpiperazinium rings of the polymers with dicyanamide anions did not undergo conformational changes in solution, in contrast to those of the polymers with chloride anions. This increased stability contributed to the preservation of the π-conjugated length and helical conformations of the polymers in solution. The polymers obtained in this study reacted with PDA, resulting in depolymerization. The results obtained in this study indicate that Zincke salts with dicyanamide anions can be used to synthesize stable ionic helical polymers under environment-friendly reaction conditions. Other polymers were synthesized in a similar manner.

## Conflicts of interest

There are no conflicts to declare.

## Supplementary Material

RA-008-C8RA05686B-s001
