# Supplementary material for: Eco-friendly synthesis of ionic helical polymers and their chemical properties and reactivity
Source: RSC Adv. 2018 Aug 24;8(52):29988–94. doi: 10.1039/c8ra05686b (PMC9085401; doi:10.1039/c8ra05686b)
Supplement: RA-008-C8RA05686B-s001 [file RA-008-C8RA05686B-s001.pdf]

Electronic Supplementary Material (ESI) for RSC Advances.
This journal is © The Royal Society of Chemistry 2018

Supporting Information

Eco-friendly Synthesis of Helical Conjugated Polymers and Their Chemical Properties and Reactivity

Isao Yamaguchi,* Yuki Tanaka and Aohan Wang

Department of Material Science, Faculty of Science and Engineering,

Shimane University, 1060 Nishikawatsu, Matsue 690-8504, Japan

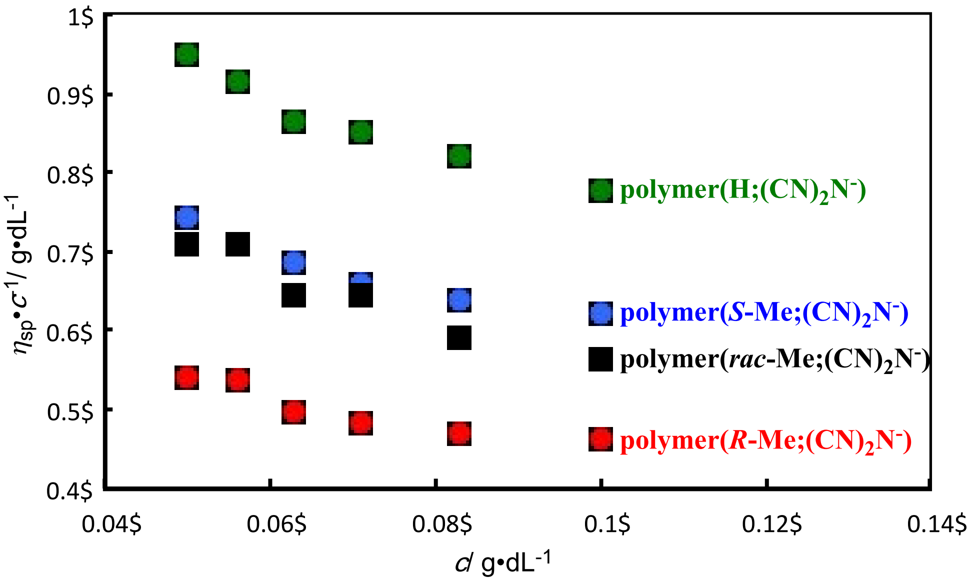

Figure S1. Concentration dependence of the reduced viscosities of the polymers in DMSO at 30 ˚C.

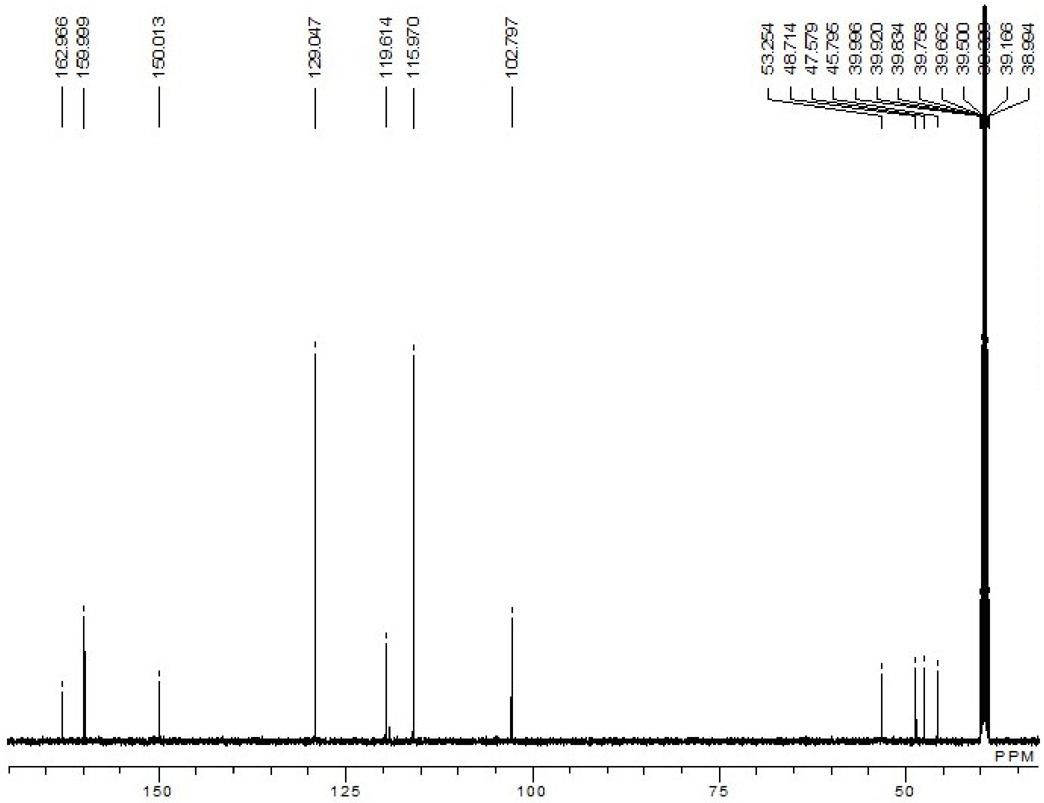

Figure S2. ^{13}C NMR spectrum of **model(H;(CN)$_2$N$^-$)** in DMSO-d_6.

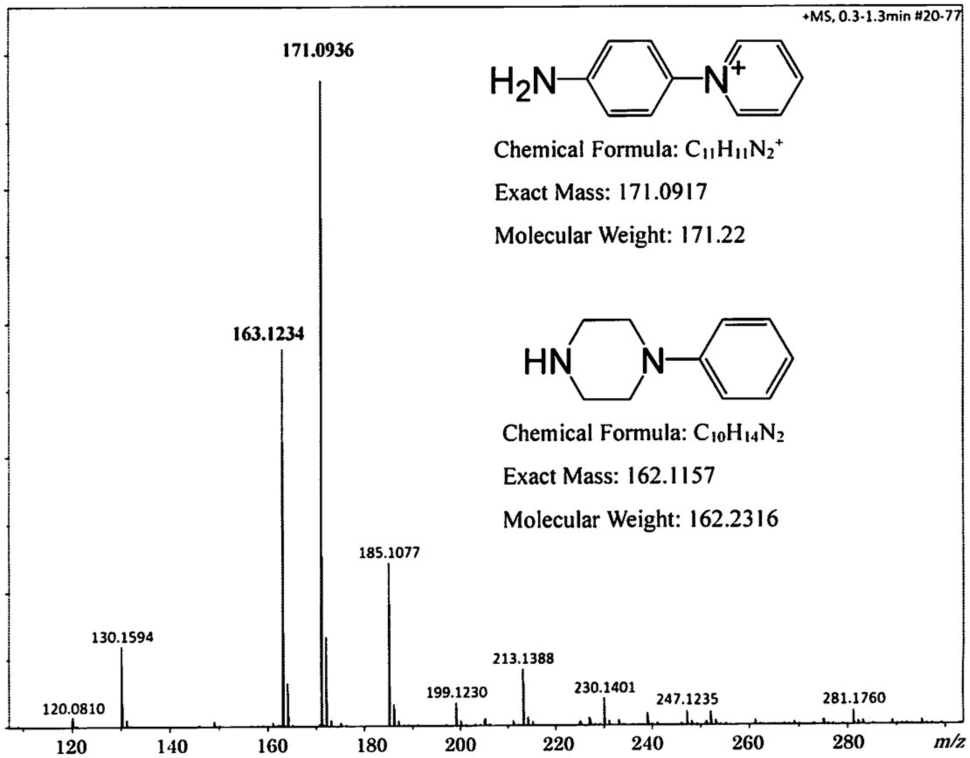

Figure S3. ESI TOF-MS spectrum of the products from the reaction of **model(H;(CN)$_2$N$^-$)** with PDA.

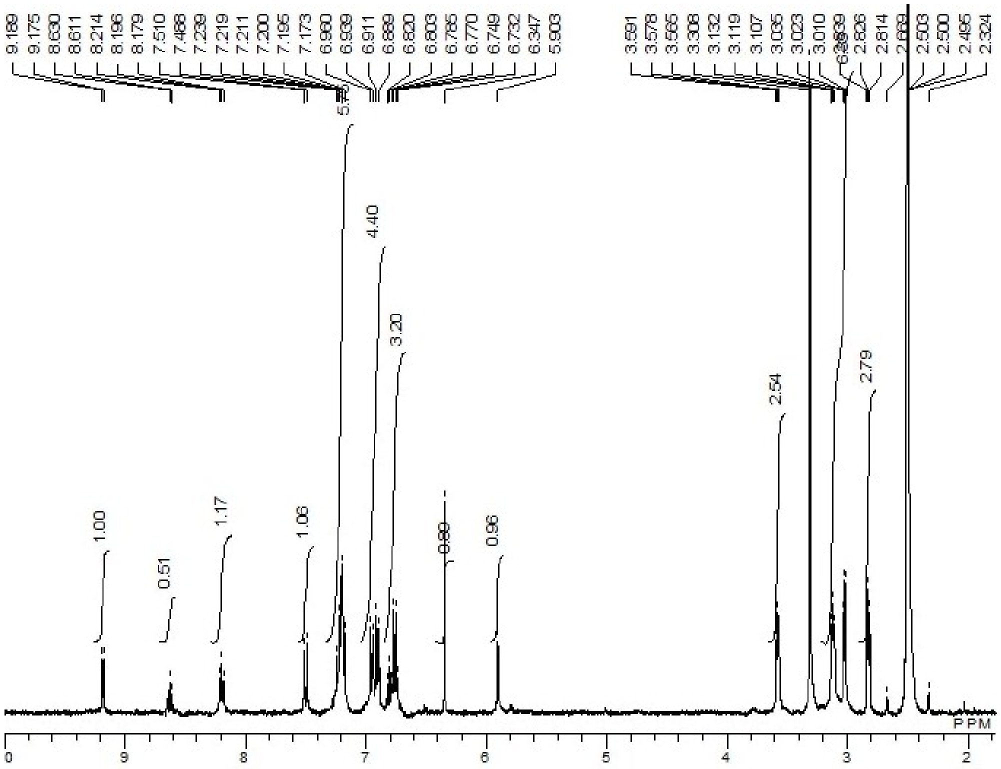

Figure S4. ^{1}H NMR spectrum of the products of the reaction of **model(H;(CN)$_2$N$^-$)** with PDA.

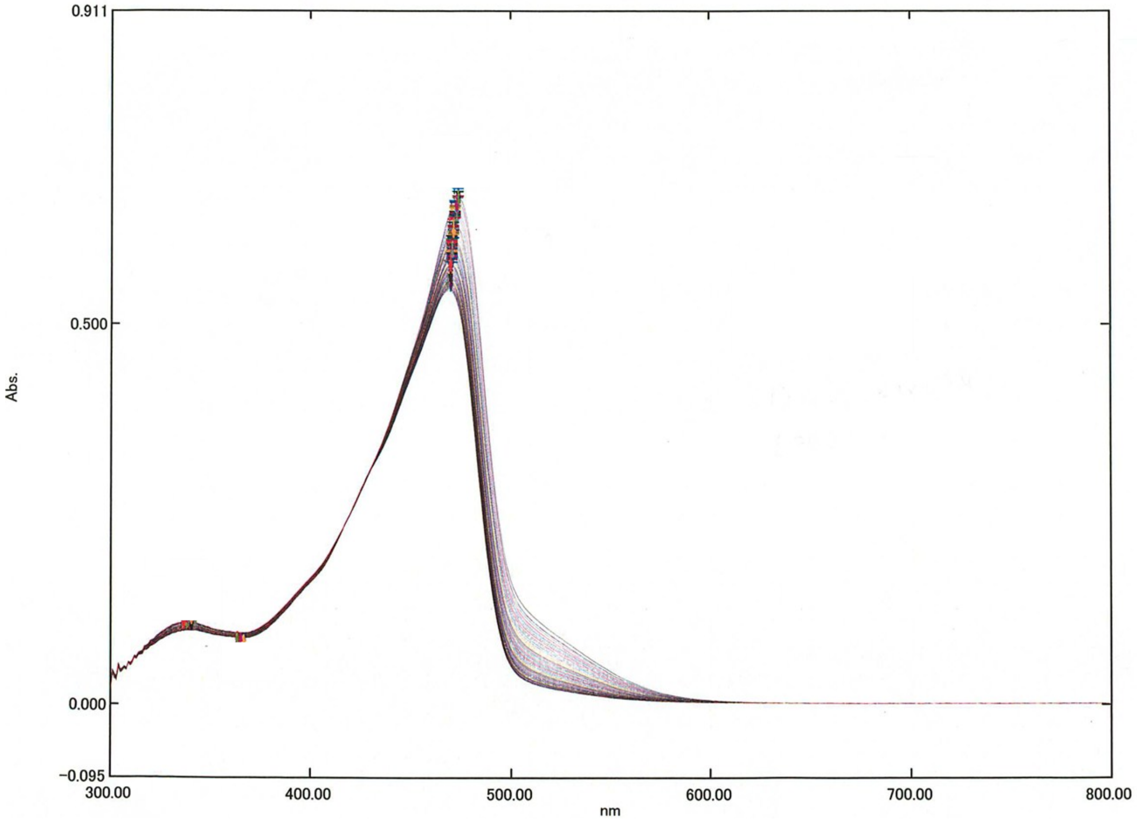

Figure S5. The UV-vis spectral changes of the DMSO solution of **polymer(H;(CN)$_2$N$^-$)** (c = 1.0 × 10^{-5} M^{-1}) in the presence of an equimolar amount of PDA.

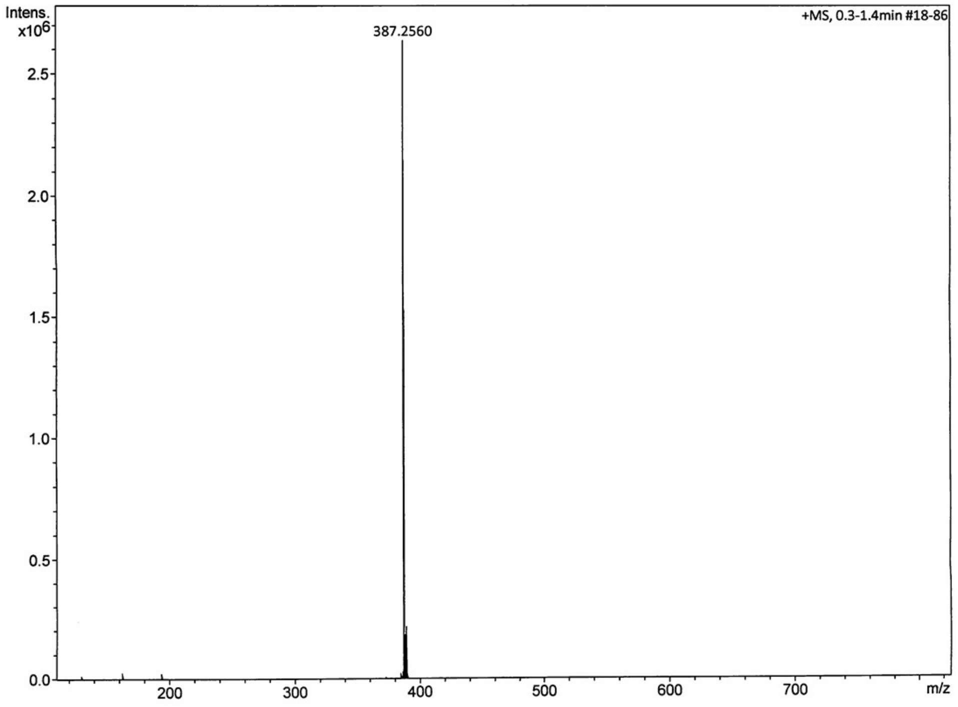

Figure S6. ESI TOF-MS spectrum of **model(H;(CN)$_2$N$^-$)** in DMSO-d_6.

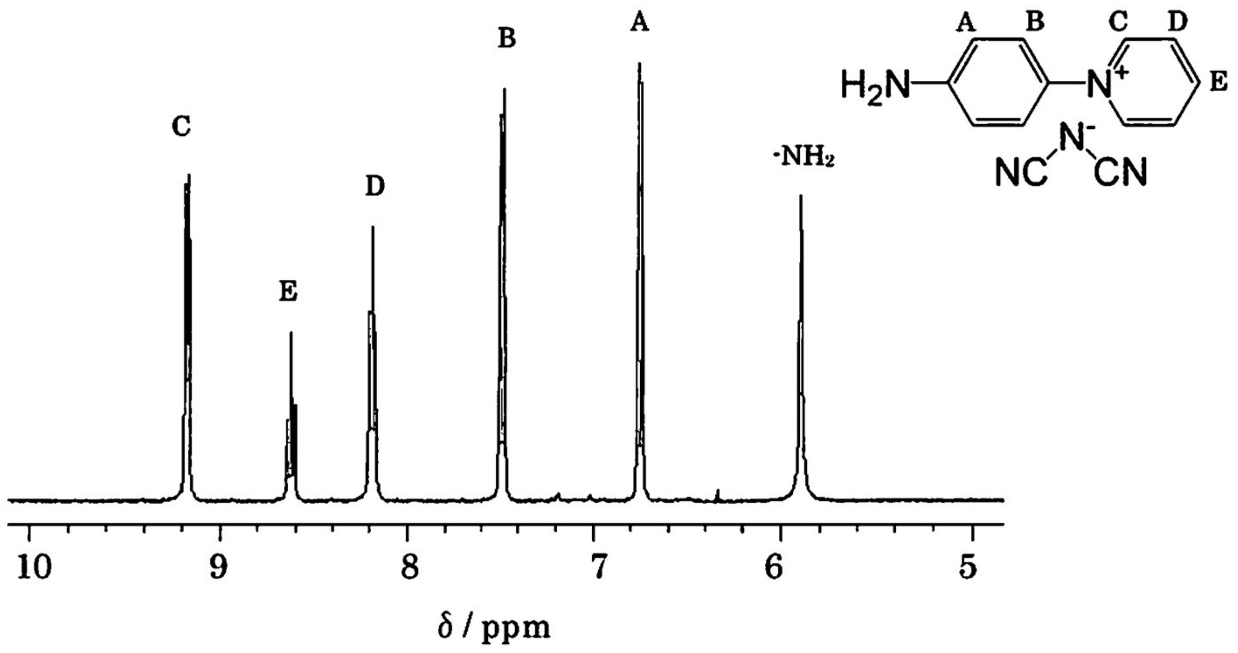

Figure S7. ^{1}H NMR spectrum of APD in DMSO-d_6.

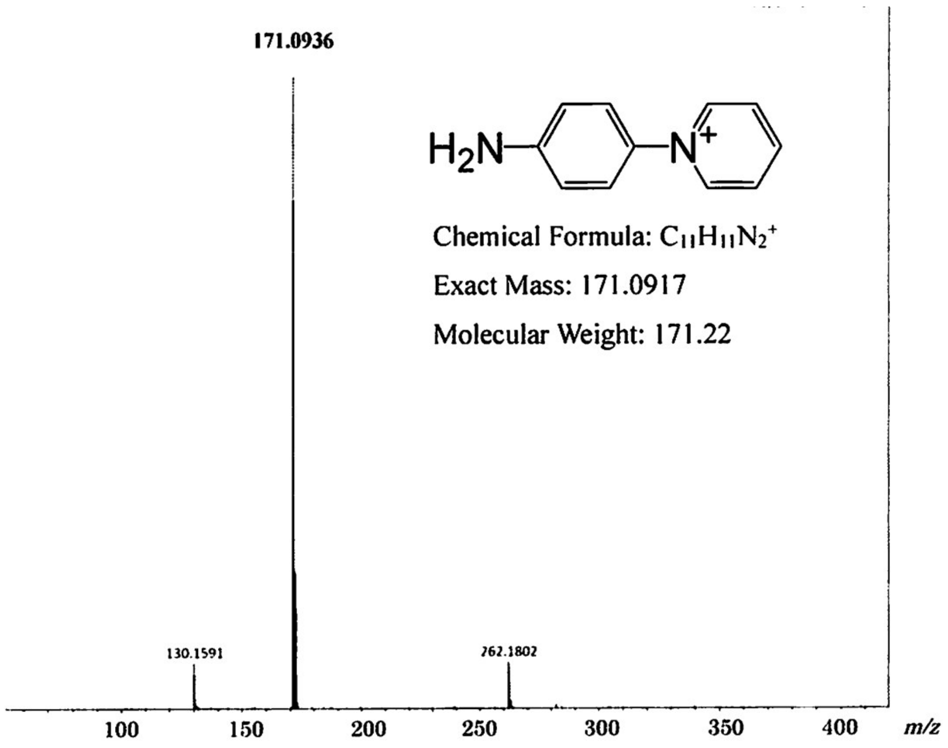

Figure S8. ESI TOF-MS spectrum of APD.

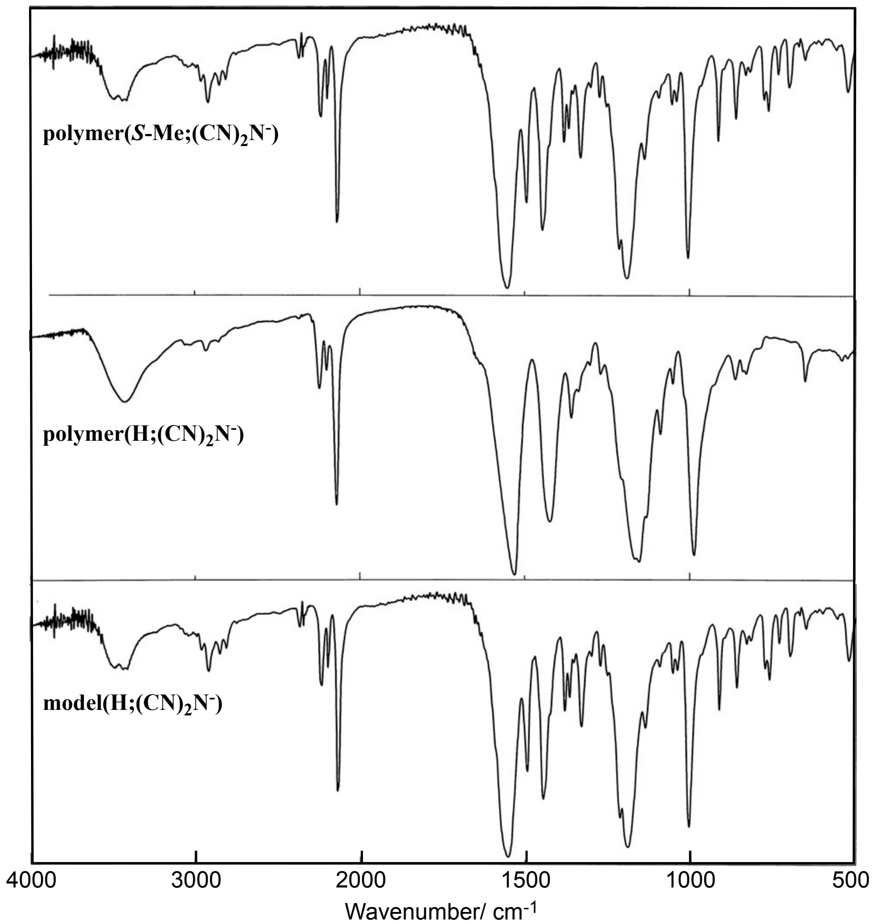

Figure S9. IR spectra of polymer(*S*-Me;$(CN)_2N^-$), polymer(H;$(CN)_2N^-$) and model(H;$(CN)_2N^-$).

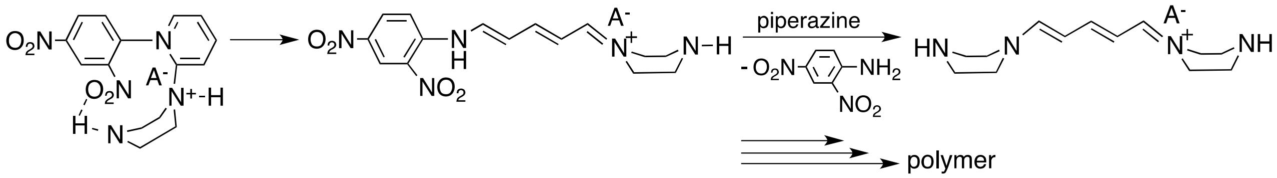

Scheme S1. Intramolecular hydrogen bond between the nitro group and the NH group..

Intramolecular hydrogen bond between the nitro group and the NH group.
